# Transcription Factor *RrANT1* of *Rosa rugosa* Positively Regulates Flower Organ Size in *Petunia hybrida*

**DOI:** 10.3390/ijms23031236

**Published:** 2022-01-22

**Authors:** Yong Xu, Yongxiang Xing, Tiantian Wei, Pengqing Wang, Yue Liang, Mengmeng Xu, Haiquan Ding, Jianwen Wang, Liguo Feng

**Affiliations:** College of Horticulture and Plant Protection, Yangzhou University, Yangzhou 225009, China; yongxu@yzu.edu.cn (Y.X.); xingyongxiang2021@163.com (Y.X.); lsghfjby2021@163.com (T.W.); wpq19980127@163.com (P.W.); liangyuecna@163.com (Y.L.); xummeng@163.com (M.X.); DHQ10108@163.com (H.D.); jwwang@yzu.edu.cn (J.W.)

**Keywords:** *Rosa rugosa*, *RrANT1*, flower size, epidermis cells, overexpression, *Petunia hybrida*

## Abstract

The flower is the main organ that produces essential oils in many plants. The yield of raw flowers and the number of secretory epidermal cells are the main factors for essential oil production. The cultivated rose species “Pingyin 1” in China was used to study the effect of *RrANT1* on floral organ development. Eighteen *AP2* transcription factors with dual *AP2* domains were identified from *Rosa rugosa* genome. *RrANT1* belonged to *euANT*. The subcellular localization results showed that *RrANT1* protein is localized in the nucleus. The relative expression level of *RrANT1* in the receptacle is higher than that in petals in the developmental stages, and both decreased from the initial phase to senescence. Compared with the *RrANT1* expression level in petals in the blooming stage, *RrANT1* expression level was significant in petals (~48.8) and highest in the receptacle (~102.5) in the large bud stage. It was only highly expressed in the receptacle (~39.4) in the blooming period. *RrANT1* overexpression significantly increased petunia flower and leaf sizes (~1.2), as well as flower fresh weight (~30%). The total number of epidermis cells in the petals of overexpressing plants significantly increased (>40%). This study concluded that *RrANT1* overexpression can increase the size and weight of flowers by promoting cell proliferation, providing a basis for creating new rose germplasm to increase rose and essential oil yield.

## 1. Introduction

The flower is an important reproductive organ, which plays a key role in plants. A typical dicot flower is composed of four homeotic structures, namely sepals, petals, stamens, and carpels, which are originated and differentiated from flower primordia. Many studies have shown that the size of floral organs is precisely regulated by a complex regulatory network [1] and is determined by the interaction between genotype and environmental influence. This size is reflected by the number and size of cells at the cellular level, which is regulated by cell proliferation and expansion during organ growth [2].

Many genes affect organ growth and development by regulating cell proliferation and expansion. *KLU* (*KLUH*) affects the enlargement of petals, leaves, and sepals [3]. *GRFs* (GROWTH-REGULATING FACTORS) and *GIFs* (GRF-INTERACTING FACTORs) act as transcription co-activators to regulate the growth and shape of petals positively [4,5]. *miR396* [6], *BB* [7], *DA1*, and *DAR1* (DA1-related) genes [8] limit the duration of the proliferation to affect the size of organs. Meanwhile, *MED8* (MEDIATOR COMPLEX SUBUNIT 8) and *BPE* (BIGPETAL) can regulate organ size positively by regulating cell expansion, but *MED25* has a negative effect [9,10]. In general, a certain compensation mechanism exists between cell proliferation and expansion. The size of plant organs mainly depends on cell proliferation as cell expansion is often limited [11].

AINTEGUMENTA (*ANT*) transcription factors (TFs) are important regulators that affect floral organ growth and development. They belong to the *AP2* subfamily in the *AP2/ERF* transcriptional regulatory family [12]. This subfamily consists of two APETALA 2/ETHYLENE RESPONSE FACTOR (*AP2*/ERF) domains and plays an important role in nutrition and reproductive organ development. The *AP2* subfamily is divided into *AP2* and *ANT* groups [13], which play different roles in the development of floral organs [14]. The *AP2* group (*euAP2*) affects the morphological transformation of various parts of the floral organs, whereas the *ANT* group regulates the development of the floral organs [15,16]. Several *ANT* group members, namely *ANT* (AINTEGUMENTA) and *AIL1-7* (AINTEGUMENTA-LIKE1-7) have been isolated from *Arabidopsis thaliana*, *Dendrobium officinale*, *Glycine max*, and *Brassica pekinensis* [17,18,19,20].

*AILs* of *A. thaliana* play a regulatory role in meristem development and participate in the development of ovules and floral organs [21]. *AIL6* overexpression also increases the size of floral organs, but it hinders cell differentiation, resulting in the loss of characteristics in the epidermal cells of sepals, petals, and stamens. Meanwhile, the meristem continues to proliferate, resulting in a sepal phenotype similar to that observed when *AIL2* was overexpressed, i.e., extremely reduced and shaped [16,22]. The ChIP-seq studies of *ANT* and *AIL6* demonstrated that both are important for early flower development in *A. thaliana* [23]. The *ant ail6* double mutant shows defects during both vegetative and reproductive development [21]. *ANT* has overlapping functions with *AIL6* in flower development, and it plays a more important role than *AIL6* in promoting floral organ growth [21,24].

*ANT*s have comprehensive roles in regulating flower organ size and number, petal cell characteristics, and flower meristem characteristics in a variety of plants. In *Petunia hybrida*, increasing and decreasing the expression of *AtANT* caused the increase and decrease of plant floral organs, respectively [25,26]. Ectopic expression of *ANT* in *A. thaliana* improved the quality of flowers threefold, indicating that ectopic expression of *ANT* can increase organ quality by enhancing organ growth. Smaller flowers and fewer outer petals and stamens were observed when *ANT* was lacking [24], and the *ant* mutant reduced the number of floral organs in *A. thaliana* [27]. The *NtAIL* gene affects the growth of plant leaves, flowers, and stems by controlling the cell proliferation and expansion in *Nicotiana tabacum*. *NtAIL* overexpression helps increase the leaf and corolla size in the transgenic *N. tabacum* [28]. Wen et al. found that silencing the *CmANT* gene led to the reduction of the average diameter of lingual inflorescence significantly, and the number of lingual and tubular flowers was also reduced in *Chrysanthemum morifolium* [29]. Ectopic overexpression of *CmoANT1.2* from pumpkin (*Cucurbita moschata*) in *Arabidopsis* increased organ size and promoted the growth of grafted plants by accelerating graft union formation [30].

Rose essential oil is extracted from the flowers of *Rosa rugosa* and is known as “liquid gold” because of its high economic value and health benefits. However, the content of the essential oil in flowers is quite limited (0.02–0.05%), which makes the oil expensive, thereby restricting the development of the related industries. Our previous cytological study on *R. rugosa* proved that the synthesis and accumulation of essential oil occur in the petal’s secretory epidermal cells [31]. In this study, we aimed to explore the candidate gene for regulating flower size by cloning, expression analysis, and the ectopical expression of *RrANT1*. This work confirmed that *ANT* positively regulates flower organ size and would promote high essential oil breeding of the cultivation of *R. rugosa.*

## 2. Results

### 2.1. Identification and Phylogenetic Analysis of RrAP2 Family

**Figure 1:**
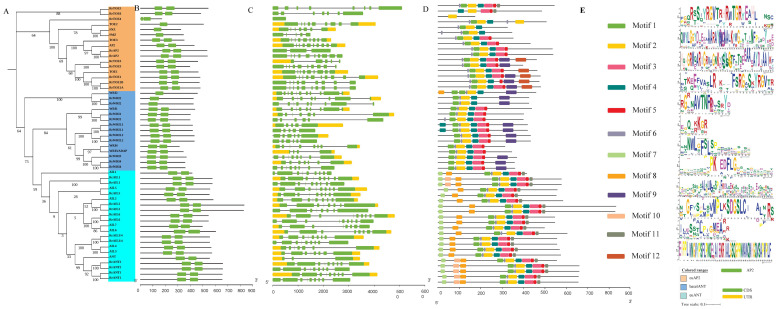
The NJ tree, *AP2* domains, gene structures, motifs, and corresponding sequences logos of ANT transcription factors of *Rosa rugosa*. (**A**) The NJ tree of *AP2* transcription factors includes *R. rugosa*, *R. chinensis*, and *Arabidopsis thaliana*, and numbers on the branch are bootstrap values. The three classes are distinguished by nodes of different colors. (**B**) The locations of the two *AP2* domains are labeled by the nucleotide scaleplate. (**C**) The exons (rectangles) separated by introns (lines) are colored yellow (untranslated region, UTR) and green (coding sequence, CDS), and their locations are labeled by the nucleotide scaleplate. (**D**) The colored boxes represent motifs whose locations are labeled by scaleplate of amino acid residue. (**E**) Motifs are listed as sequence logos.

To investigate the phylogenetic analysis of the *RrAP2* family, all the 18, 16, and 18 *AP2* TFs with dual *AP2* domains were identified from the genomes of *R. rugosa, R. chinensis*, and *A. thaliana*, respectively. Their names were numbered according to the top BLASTP search of known *AtAP2* TFs (Table S1). In addition, isoforms (putative alternative splicing *AP2s* mRNA) were distinguished by number suffix, and homologous *AP2s* were distinguished by uppercase letters suffix. The phylogenetic tree was constructed using the full-length protein sequences of all *AP2s* belonging to three groups (7 *euAP2*, 5 basel*ANT*, and 6 *euANT*), referring to the known topological structure of *AP2* family [13] (Figure 1A). *AP2* can be speculated to be highly conserved. Almost all *AP2*s in *R. rugosa* and *R. chinensis* belonging to basel*ANT* and *euANT* were adjacent to each other in pairs, which indicated that *RrAP2*s and *RcAP2*s had stronger congruent relationships. Two *RrANT*s (i.e., *RrANT1* and *RrANT2*) existed in the *euANT* group.

The *AP2* subfamily contained two *AP2* domains (Figure 1B). Most of these genes in the same group had the same exon–intron pattern, especially in the *euAP2* group, in which the position, number, and length of the exons and introns were very similar (Figure 1C). Twelve significantly enriched motifs were highly conservative (Figure 1E). The top five motifs arranged in 1-2-4 and 3-5 were conserved in the three groups and should overlap with the two *AP2* conserved domains (Figure 1D). Other unique motifs obviously separated the groups. For the euANT group, the insertions in the pre-domain region for extra motifs 7 and 8 separated from the basalANT lineage without the motifs [23].

### 2.2. RrANT1 Identification and Characterization

The expressions of *RrANT1* and *RrANT2* in the petal were first analyzed. The expression level of *RrANT2* was relatively low, especially after the red-blooming stage. Here, *RrANT1* was studied in this study.

**Figure 2:**
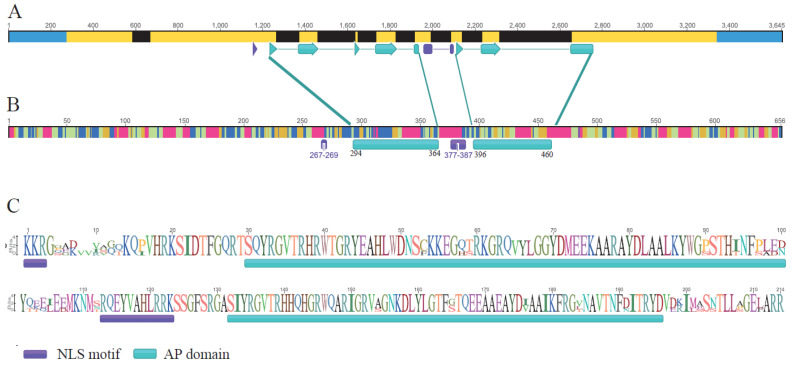
The gene structure, conserved sequences, and the predicted amino acid residues of *RrANT1*. (**A**) The exons separated by introns are colored blue (untranslated region, UTR) and yellow (coding sequence, CDS). (**B**) The length and location of the two AP domains (green). (**C**) The predicted amino acid residues and nuclear localization signal (NLS) (purple).

The obtained 3′ and 5′ of cDNA sequence fragments amplified by the RACE method were about 500 and 2200 bp, respectively. The full-length cDNA sequence spliced by the two fragments was 2259 bp (MF802281.1) and included a 1917 bp coding sequence (CDS), a 275 bp 5′ untranslated region (UTR), and a 301 bp 3′ UTR. The expressed region was separated by eight introns (Figure 2A). The conserved middle region consisted of two AP domains and a nuclear localization signal (NLS) found through multiple sequence alignment (Figure 2B). The gene structure was identified as *R. rugosa ANT* (*RrANT1*, LOC112169406) gene structure. Theoretically, it encoded a polypeptide of 656 amino acid residues (aa) with 72.384 kDa molecular weight, and the isoelectric point was 7.46. The *RrANT1* protein contained 46.83% of alpha helices, 15.67% of extended strands, 8.17% of beta turns, and 29.33% of random coils (Figure 2C).

### 2.3. Relative Expression Level of RrANT1 in the Flower Tissues and Leaves of R. rugosa

**Figure 3:**
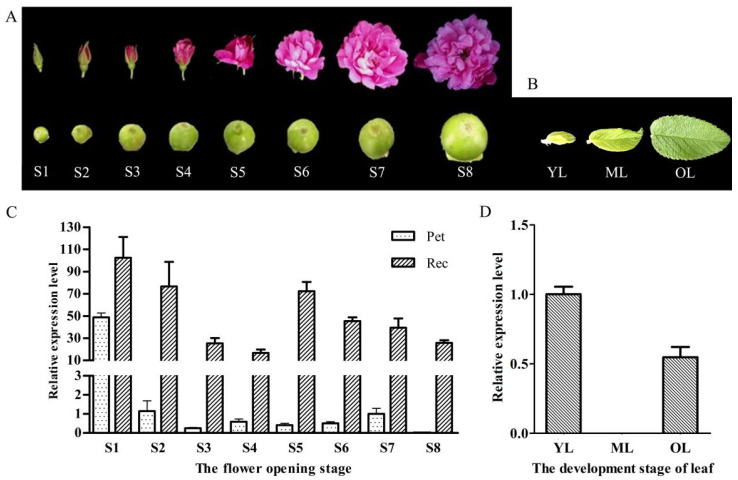
Transcript abundance of *RrANT1* in petals, receptacles, and leaves at different development stages. (**A**,**C**) S1: Large bud stage, S2: Reddish stage, S3: Flowering initiation stage, S4: Flower bud half-opening stage, S5: Initial opening stage, S6: Semi-opening stage, S7: Full opening stage, S8: Withering stage. (**B**,**D**) OL: Old leaf, ML: Mature leaf, YL: Young leaf.

To compare the expression level of *RrANT1* in the flower tissues and leaves of *R.* *rugosa* at different stages (Figure 3A,B), qRT-PCR was chosen for gene expression analysis. The relative expression levels for the tissues of the flower were compared with the expression level of petals in the blooming stage (S7). For the petals, *RrANT1* was highly expressed only in the large bud stage (S1) (~48.8), whereas the expression levels in the remaining seven stages were very weak. Almost no expression was found in the decay stage (~0.0). The expression level levels of *RrANT1* in the receptacle during the eight opening stages of rose were detected. The level declined from the large flower bud stage (S1) (~102.5) to the half-blooming stage of the flower bud (S4) (~16.7). Then, it increased sharply in the early blooming stage (S5) (~72.3), almost reaching the red-blooming stage (S2). Afterward, it gradually decreased to the withering stage (~25.8) (Figure 3C). For the leaves at the three growth stages, the expression level of *RrANT1* was similar to that in petals. Compared with the expression level of young leaves, almost no expression was found in mature leaves (~0.0), and a low relative expression level was found in old leaves (~0.4) (Figure 3D). The results showed that the relative expression level of *RrANT1* in the initial stage of organ development was much higher than that in the mature stages whether in petals, receptacles, or leaves.

**Figure 4:**
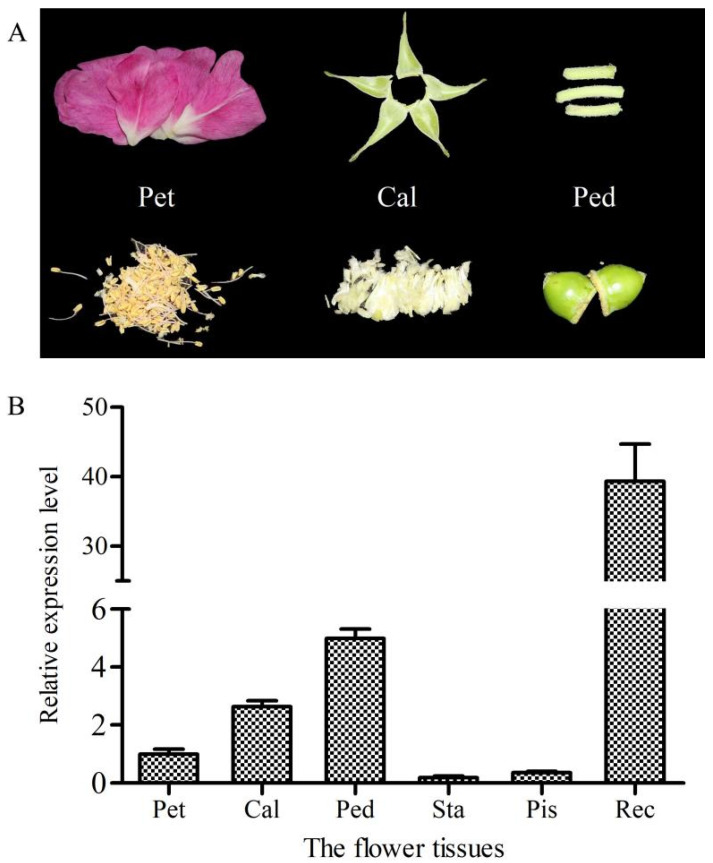
Transcript abundance of *RrANT1* in tissues of the flower in the blooming stage. (**A**) The samples of six flower tissues. (**B**) Relative expression levels in tissues. (**A,B**) Pet: Petal, Cal: Calyx, Ped: Pedicle, Sta: Stamen, Pis: Pistil, Rec: Receptacle.

Furthermore, the expression level of *RrANT1* in various parts of the floral organs (Figure 4A) in the blooming stage (S7) was also detected. The receptacle had the highest expression level (~39.4). Trace expressions were found in the calyx and pedicle, i.e., 7.1% and 7.7% of the that in the receptacle, respectively. Almost no expression was found in the stamens, pistils, and petals (Figure 4B).

### 2.4. Subcellular Localization of RrANT1

**Figure 5:**
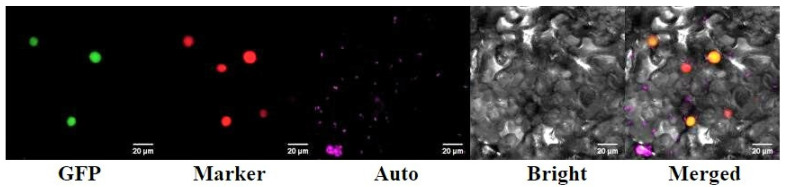
The subcellular colocalization of *RrANT1* in the lower epidermis of the tobacco leaves. GFP: the fluorescent signal of *RrANT1*-GFP; Marker: the red fluorescence signal of chloroplast DNA marker; Auto: fluorescence signal of chloroplast; Bright: bright-field image; Merged: merged images of bright-field and green fluorescence images.

The confocal laser scanning microscope was used to observe the cellular localization of the *RrANT1*-GFP fusion protein in the lower epidermis of tobacco leaves. The superposition of green fluorescence of *RrANT1*-GFP fusion protein and the red fluorescence of marker indicated that *RrANT1* was located in the nucleus (Figure 5). It showed that *RrANT1* was a nuclear localization protein and was consistent with the subcellular localization prediction result.

### 2.5. Phenotypic and Microstructure Observation of Leaves and Flowers of Transgenic Petunia

**Figure 6:**
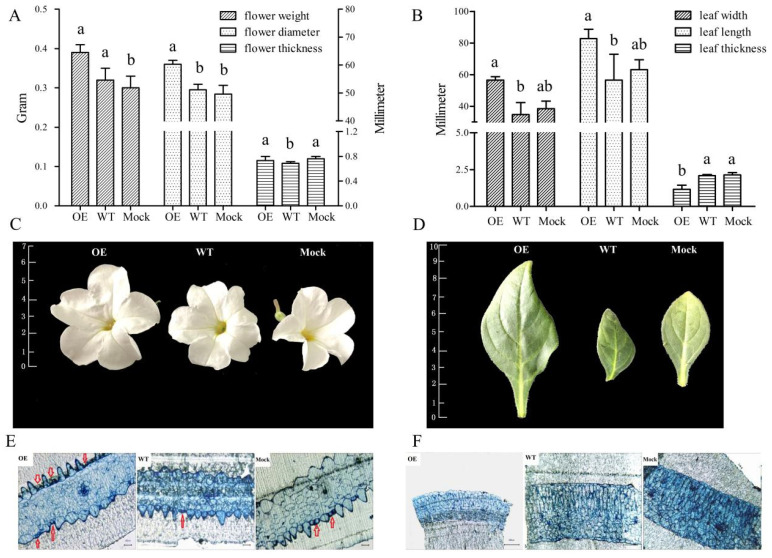
Morphological comparative analysis and microstructure observation of the flower and leave between the OE and control groups in the blooming stage. (**A**) The diameter, thickness, and weight of the fresh flowers. (**B**) The width, length, and thickness of the leaves (a and b represent the significance of the difference). (**C**,**D**) The morphology and size of the flowers and leaves. (**E**,**F**) The shape and arrangement of cells of the semi-thin slice of petal and leaf stained by toluidine blue. Ticks interval = 1.0 cm.

To study the *ANT* gene function in petunia, transgenic plants were obtained. Our analysis showed that, compared with the control group, the size of the flowers and leaves of the OE group significantly increased. The diameter of the petal of the OE increased by about 1.2 times, but the thickness did not change significantly. The weight of the fresh flowers also increased significantly (~30%) (Figure 6A). The length and width of the leaves of the OE were 2.1 and 1.7 times those of the WT, and 1.4 and 1.5 times those of the Mock, respectively. However, the thickness of the leaves was only about one half of that in the control groups, possibly because the leaves grew faster (Figure 6B). However, leaves reached the normal thickness afterward, thereby indicating that the developmental period of the leaves of OE may be delayed compared with that of the control groups. The shapes of the leaves and flowers (Figure 6C,D) and the number of flowers were not obviously different between the OE and control groups.

Regarding the semi-thin slice, many incompletely differentiated cells were observed in the petal epidermis of OE, especially in the lower epidermis (Figure 6E, as indicated by red arrows). These cells were small and had a dense cytoplasm, and they were stained by toluidine blue. The surrounding cells that were differentiated had a high degree of vacuolization. In the upper epidermis, although the number of mature cells of OE (~47%) was slightly lower, the number of cells that were not fully differentiated (~53%) was significantly more than that of the control groups. In the lower epidermis, the number of the two kinds of cells increased compared with the control groups, i.e., ~2.4 and ~1.5 times of Mock and WT, respectively. Therefore, compared with control plants at the blooming stage, the total number of the epidermis cells in the petals of OE plants significantly increased, i.e., ~1.8 and ~1.4 times of Mock and WT, respectively. In addition, the size of the petal epidermal cells of OE was not significantly different from that of the control plants, as observed from the cross-sectional view. However, the layers were fewer, and the cells were arranged loosely in a fence organization in the OE group, as observed using the leaf cross-section (Figure 6F). This finding was consistent with the decrease in the leaf thickness of the OE plants.

## 3. Discussion

The *ANT* gene belongs to the *AP2* subfamily of plant-specific transcription factors. The *AP2* subfamily of the three groups (*euAP2*, basel*ANT*, and *euANT*) exhibited similar patterns in gene and domain duplication, exon arrangement, according to the phylogenetic analysis of the *AP2* family. The five motifs arranged in 1-2-4 and 3-5 were conserved in the three groups and should overlap with the two AP2-conserved domains, which shows that *AP2* domain was duplicated prior to the divergence of the two major lineages of *euAP2* and *ANT*. Although it has a highly conserved *AP2* domain, there are differences in the amino acid sequence of other regions, suggesting the importance of the *AP2* domain and the diverse functions of *ANT* group members. Within the *ANT* lineage, the *euANT* lineage is characterized by four conserved motifs: one insertion in the AP2-R1 domain (motif 6) and three in the predomain region (motif 7, motif 10, and motif 8) [13]. Much research has shown that genetic redundancy exists among members of *euANT* family [16,21,23,32], which may be associated with their special structure, i.e., the same DNA binding motifs. The AP2-R1 domain of *RrANT1* Contains VYL modification sites related to transcription activity, which indicates that the *RrANT1* may be similar to *AtANT*, having a transcription regulation function [33].

*ANT* can perform an array of functions including the establishment of the floral meristem, the specification of floral organ identity, and the growth and morphogenesis of developing floral organs (such as sepals, petals, stamens, and carpels) [21,23]. *RrANT1* was expressed significantly in the large flower bud stage of *R. rugosa* in this study, especially in petals and receptacles, indicating that it should regulate the development of floral organs [29]. As the cells in the large flower bud are still in the early stage of development, the high expression of *RrANT1* can promote rapid cell proliferation. In the reddish stage, cell proliferation almost stopped in the petal, and the expression level of *RrANT1* decreased rapidly and was maintained at a low level. The expression level of *RrANT1* in the receptacle was maintained at a high level in the early stage and decrease after the initial receptacle was formed. The similar expression pattern of *RrANT1* in different developmental stages of leaves showed that *RrANT1* was also involved in the growth of the leaves. The results of the study proved that *ANT* has age-dependent expression, and it is expressed primarily in young actively dividing tissues [32]. In addition, the study also showed that the expression of *RrANT1* in the receptacle was always higher than that in the petal at different flower development stages except for the large flower bud stage. This finding may be because that the growth rate of the receptacle is slower than that of the petal, and the receptacle needs to maintain cell proliferation [34]. Besides, the receptacle is the place where the rosehip is produced, and a high expression of *RrANT1* is required to promote the development of the inner part of the ovule [34,35]. The expression was relatively high in the receptacle, whereas it was extremely low in other parts of floral organs in the blooming period, as *ANT* expression then becomes more restricted to particular regions within developing petals, stamens, and carpels [36]. This result proved that *RrANT1* expression has significant temporal and spatial differences during development.

The ectopically expressed *RrANT1* caused increased organ size with larger petals as a result of cell division and cell expansion. Some epidermal cells of petals were still in the early stages of development and appeared to extend the period of cell division. The reason for this phenomenon should be that *ANT* regulates cell proliferation and organ growth by maintaining the meristematic competence of cells during organogenesis [21,34]. There is potential coordination between cell division and expansion to maintain a certain organ size [11], and that is why the number of petal epidermis cells of OE was more, while the fully differentiated ratio of those was lower. Besides, the mature cells of petal epidermis cells of OE were normal. This suggests that the *ANT* positively promotes cell proliferation to increase the size of the flower [37]. Petal and leaf might be considered separate organs in terms of genetic modification when it comes to size [26]. *ANT* may directly regulate potential leaf-growth-related target genes, such as ANGUSTIFOLIA3 (*AN3*), which is a transcriptional activator of the *GIF* family and is involved in leaf growth, and ectopic overexpression of *AN3* resulted in enlarged leaf size [4,38].

It has been studied that *ANT*-binding sites are associated with genes involved in meristem and flower organ development. These include class B and C floral homeotic genes, and genes involved in growth regulatory and vascular development [25]. Besides, maize *ANT1* is also proved to be a master switch that functions upstream of key regulators of vascular and vein development, chloroplast development, and photosynthesis through its target genes [39]. Additional work is needed to further elaborate the roles of *ANT* and its direct target genes in both these developmental processes and plant floral organogenesis. In all, it is indicated that *ANT* is sufficient for floral organ growth, and that increasing organ mass by ectopic *ANT* expression should be a new powerful method for improving the yield of plants with larger flowers and leaves. The overexpression of the *RrANT1* gene could be adopted to increase the number of epithelial secretory cells, thereby promoting the size and the fresh weight of rose flowers. It should be that the bigger flower would increase flower yield per unit area of rose planting field and more the raw materials would produce more essential oil. The correlation between flower size and oil content should be proved based on the 35s: *ANT* or *ant* lines. Due to the inefficient genetic transformation of the woody rose, our lab is working to obtain transgenic lines by somatic embryo transformation to verify the function and molecular mechanism of *RrANT1* in *R. rugosa* definitively.

## 4. Materials and Methods

### 4.1. Plant Materials

The cultivated species *R. rugosa* “Feng Hua”, also named “Pingyin 1”, was planted in the rose resource planting field in Yangzhou University (N 32°23′27.64′′, E 119°25′10.23′′) under natural conditions with regular irrigation. The three-years-older cutting seedlings were selected as materials. To investigate the changes of the transcript abundance of *RrANT1* during the opening stage of rose, eight periods, S1–S8, were named as follows: large bud stage (S1); reddish stage (S2); flowering initiation stage (S3); flower bud half-opening stage (S4); initial opening stage (S5); semi-opening stage (S6); full opening stage (S7); withering stage (S8) (Figure 2A). Both petals and receptacles were collected in each stage above. The petal, calyx, pedicle, stamen, and pistil in the full opening stage of the flower and the leaves at the young, mature, and old ages were also collected. For each sample, three replications were randomly selected from three different plants that were healthy and of the same age. The sample collection time was from 6:00–7:00 in the morning. The samples were frozen in liquid nitrogen upon collection from plants and stored in an ultra-low temperature refrigerator at −80 °C for further analysis.

The tissue culture seedlings of *P. hybrida* ‘Mitchell Diploid’ were used as the genetic transformation receptor for the overexpression of *RrANT1*. The culture condition for *N. tabacum* and *P. hybrida* used in this study is set with sunlight period for 16 h at 22 °C, and dark period for 8 h at 18 °C in artificial climate incubators.

### 4.2. Identification, Phylogenetic, Gene Structure, and Motif Composition Analysis of AP2 Family

To identify potential *RrAP2s*, the Hidden Markov Model profile of the *AP2* domain (Pfam number PF00847) was used for searching candidate genes in the whole genome of *R. rugosa* (http://eplantftp.njau.edu.cn/Rosa_rugosa/, accessed on 20 September 2021) to identify all *RrAP2* by HMMER v3.3.2 [40,41]. The candidate *AP2* genes were checked in the Conserved Domain Database (CDD, https://www.ncbi.nlm.nih.gov/, accessed on 21 September 2021) and Pfam database (http://pfam.xfam.org/, accessed on 21 September 2021) [40,42]. All members of the *RcAP2* family were identified from the genome of *R. chinensis* (https://www.rosaceae.org/, accessed on 22 September 2021) by the same method. *AtAP2s* were downloaded from the PlantTFDB (http://planttfdb.gao-lab.org/, accessed on 24 September 2021). A neighbor-joining (NJ) tree of protein sequences of *RrAP2*s, *AtAP2*s, and *RcAP2*s was built by MEGA-X (https://www.megasoftware.net/, accessed on 26 September 2021) with p-distance and pairwise deletion parameters [43,44]. The 1000 bootstrap replications were used to verify the reliability of the phylogenies, and the online tool ITOL (https://itol.embl.de/, accessed on 27 September 2021) was used for coloring [45].

The conserved domain data of the *RrAP2* protein sequence were obtained through the website of NCBI Batch CD-search (https://www.ncbi.nlm.nih.gov, accessed on 28 September 2021). The exon and intron distribution data were obtained according to Rose’s *GFF* gene annotation file. The motifs were predicted by the online tool MEME 5.3.3 (https://meme-suite.org/meme/, accessed on 28 September 2021) with default parameters. The gene structures and motifs were visualized by Tbtools 1.086 [46].

### 4.3. The Extraction of Nucleic Acid

The DNA and RNA of all the samples in this study were obtained as follows: the genomic DNA was extracted from young leaves from *R. rugosa* via the DNeasy Plant Mini Kit (QIAGEN, Dusseldorf, Germany). The total RNA of different samples was extracted via MiniBEST Universal RNA Extraction Kit (TaKaRa, Tokyo, Japan) according to the manufacturer’s instructions. The samples included the flower and its tissues, as well as leaves of *R. rugosa*, introduced in Section 4.1, and the young leaves from the three groups of petunia plants (OE, Mock, and WT) shown in Section 2.5.

### 4.4. Cloning of Full-Length cDNA of RrANT1

Full-length cDNA of *RrANT1* was cloned by the rapid amplification of cDNA ends (RACE). For 3′ RACE, the cDNA template was synthesized by 3′ full RACE Core Set (TaKaRa, Tokyo, Japan). Gene specific outer and inner primers were designed according to the middle region of the *RrANT1* gene (Table S2). The 3′ end cDNA was amplified using nested PCR. The first round PCR (outer PCR) was processed by denaturing the cDNA at 98 °C for 1 min, followed by 25 cycles of amplification (98 °C for 10 s, 55 °C for 10 s, and 72 °C for 5 s) and extension at 72 °C for 3 min. The outer PCR product was used as the template of the second round PCR (inner PCR), using the same method for the outer PCR. The product was cloned into the pEASY-T5 Zero Cloning vector (TransGene Biotech, Beijing, China) for sequencing. For 5′ RACE, the gene specific primers were designed based on the 3′ cDNA end sequence. The 5′ RACE cDNA template was synthesized by SMARTer@RACE cDNA Amplification Kit (TaKaRa, Tokyo, Japan).

Subclone and sequencing of PCR products were in accordance with the methods used in a previous study. The assembly of 5′ cDNA end and 3′ cDNA end was obtained using pEASY-T5 Zero Cloning Kit (TransGene Biotech, Beijing, China), which represented the *RrANT1* reverse transcribed from mRNA. After testing by 1% (*w*/*v*) agarose gel electrophoresis, the *RrANT1* PCR products were cloned into the pCE2 TA/Blunt-Zero Vector and sequenced.

### 4.5. Reverse Transcription Reaction and RrANT1 Expression Analysis

The α-tubulin gene (AF 394915.1) of rose was used as the reference gene. The primers of the *RrANT1* and α-tubulin genes are listed in the supplementary file (Table S3a). For reverse transcription reaction, the cDNA of each sample was reverse transcribed from 1 μg RNA using PrimeScript RT reagent Kit (TaKaRa, Tokyo, Japan). The expression levels were quantified by real-time quantitative reverse transcription-PCR (RT-qPCR). The 25 μL PCR reaction system contained 12.5 μL SYBR^®^ *Premix Ex Taq* (2×) (TaKaRa, Tokyo, Japan), 1 μL of each primer (10 uM), 2 μL cDNA template, and 8.5 μL ddH_2_O. The amplification procedure was performed by an initial incubation of 5 min at 95 °C, followed by 40 cycles of 15 s at 95 °C, 30 s at 54 °C, and 30 s at 72 °C on CFX96 Real-Time PCR detection system (Bio-Rad, Des Plaines, IL, USA). Based on the threshold cycle (Ct) values generated from the CFX Manager software (Bio-Rad, Des Plaines, IL, USA), the expression level was calculated by the comparative Ct using the relative quantification method (2^−^^△△Ct^) [29]. The expression level of *RrANT1* in those collected tissues (Section 4.1) was analyzed.

### 4.6. Vector Construction and Subcellular Localization of RrANT1

Reverse transcription synthesis of the first strand of cDNA from total RNA used the same method described above in subSection 4.5, and the target gene fragment *RrANT1* (Figure S1) was amplified by PCR. Then, *RrANT1* connected with the vector of pBWA(V)HS-GLosgfp using gene-specific primers (Table S3b) to form the expression vector pBWA(V)HS-*RrANT1*-GLosgfp using enzyme digestion technology. The plasmid was extracted using AxyPrep^TM^ Plasmid Minniprep Kit 50-prep (Axygen) using the kanamycin-screened positive *Escherichia coli.* The plasmid was transferred into the competent cells of *Agrobacterium tumefaciens* LBA4404 by the liquid nitrogen freeze-thaw method. The plasmid of the nuclear signal marker (mKate2, a kind of red fluorescent protein) was also transferred into other LBA4404. The two kinds of cultured *A. tumefaciens* mentioned above were resuspended with the liquid of MgC1_2_ with added acetosyringone until the OD_600_ value reached 0.6 to make the infestation liquid. Afterward, they were co-injected equivalently to the lower epidermis of the leaves for one-month-old tobacco (*N. tabacum*). When the tobacco plants were cultured at low light for 48 h, the fluorescence of the cells in the infected leaf was imaged using a confocal laser-scanning microscope (Olympus FV10 ASW).

### 4.7. Overexpression (OE) of RrANT1 in P. hybrida and Microstructural Observation

The binary expression vector of pCAMBIA1304 containing *RrANT1* was constructed using the In-Fusion^®^ HD Cloning Kit (Table S3c). The PCR amplification of *RrANT1* containing restriction sites with the PCR primers was performed using PrimeSTAR^®^ HS DNA Polymerase (TaKaRa, Tokyo, Japan) based on the plasmid of *RrANT1*.

The pCAMBIA1304 was used for the control group (Mock) and the pCAMBIA1304: *RrANT1* was used for OE lines. All constructs were transformed into the competent cells of *A*. *tumefaciens* EHA105 by the liquid nitrogen freeze-thaw method, respectively. The methods of genetic transformation, tissue culture, and identification of OE established were as follows. (1) Cut the leaves of the tissue culture seedlings into small squares of about 0.5 cm × 0.5 cm in a confined environment and position the ventral surface of the leaves on the preculture medium for culturing for 2 days at 24–28 °C until the edges of the blade cuts were slightly swollen. (2) Place the pre-cultured leaves in the infection solution to sway for 5 min. (3) Place them in a co-culture medium after blotting the bacteria on the surface and culture in the dark for 2 days. (4) Transfer the leaves to the selection and differentiation medium for callus resistance screening and change the medium every 2 weeks. (5) Cut the sprouted buds and culture them in the screening rooting medium; observe the rooting situation after 2 weeks. (6) When the rooted petunia plants grew to the height of the bottle mouth, move them out to the artificial climate incubator. The different kinds of genetic transformation mediums were listed in Table S4. The identification of the transgenic petunia was performed by GUS dyeing of petunia callus and PCR detection using the designed primers (Table S3d).

To identify whether *RrANT1* was successfully transferred into petunia or not, two experiments were conducted. Firstly, the DNA was extracted from young leaves of both transgenic wild petunias, and then ~750 bp hygromycin B (Hyg) resistance gene fragments of the plasmid were amplified by PCR with the designed primers (Table S3e). The products were tested by agarose gel electrophoresis to differentiate the petunias that *RrANT1* have been integrated into the petunia genome (Figure S2A). Secondly, semi-quantitative detection for flowers at the blooming stage was adopted for the transgenic petunias screened above. The expression of *RrANT1* can illustrate its transferred state, referring to the benchmark expression level of the internal reference gene of *ubiquitin* (Figure S2B). For the biological characteristics observation of the transgenic petunia during growth, the plant morphological characteristics, such as the size and weight of the flowers, leaves, stems, and seeds, were considered. The microstructures of leaves and petals were observed by microscopy. The methods used for obtaining the semi-thin slices and observing the pieces by 2 mm × 1 mm were described in detail by Zang et al. [31].

## 5. Conclusions

The *RrANT1* transcription factor, which belongs to *euANT* and located at the nucleus, is relatively conservative. The expression level of *RrANT1* is age-dependent, meaning that it is highly expressed in the early dividing stage of petals, receptacle, and leaf, but weakly expressed during organ maturity and senescence. It proves that ectopic expression of *RrANT1* in petunia controls petal size by regulating the number and extent of cell division during organogenesis. *RrANT1* can increase the size and weight of flowers and leaves by mainly promoting cell proliferation, thereby providing a basis for creating new rose germplasm to increase the yield of rose and essential oil.

## Data Availability

Data are contained within the article or Supplementary Materials.

## Supplementary Materials

The following supporting information can be downloaded at: https://www.mdpi.com/article/10.3390/ijms23031236/s1.
