# Supplementary material for: Transcription Factor RrANT1 of Rosa rugosa Positively Regulates Flower Organ Size in Petunia hybrida
Source: Int J Mol Sci. 2022 Jan 22;23(3):1236. doi: 10.3390/ijms23031236 (PMC8835453; doi:10.3390/ijms23031236)
Supplement: Supplementary file 1 [file ijms-23-01236-s001.zip › ijms-1526575-supplementary.pdf]

Supplementary Materials

Table S1 The gene sequences and names of *RrAP2* family

Sequence	Gene name
evm.model.Chr6.4678	RrAIL1
evm.model.Chr4.20	RrAIL2
evm.model.Chr5.468	RrAIL3/4
evm.model.Chr6.2418	RrAIL6
evm.model.Chr2.276	RrANT1
evm.model.Chr6.2219	RrANT2
evm.model.Chr3.1277	RrWRI1
evm.model.Chr6.6474	RrWRIL1
evm.model.Chr1.4006	RrWRIL2
evm.model.Chr6.3660	RrWRI4
evm.model.Chr4.2960	RrWRI2
evm.model.Chr6.1996	RrAP2
evm.model.Chr5.1758	RrTOE1A
evm.model.Chr5.2157	RrTOE1B
evm.model.Chr4.1706	RrTOE3
evm.model.Chr6.1999	RrTOE4
evm.model.Chr4.2805	RrTOE5
evm.model.Chr4.2818	RrTOE6

Table S2 The primers used for amplifying the *RrANT1* by RACE

Gene name	Primer(5′-3′)	Application
RrANT	TTCCGTGGGGTAAATGCG	1st of 3′ RACE
RrANT	TTCAAATCCTTCGTCGCT	2nd of 3′ RACE
RrANT	CCAAGCAGCAAATAGAGGCAAGTGAGACA	5′ RACE

Table S3 The primers used for different kinds of experiment

Coding	Experiment	Gene	Forward Primer (5'-3')	Reverse primer (5'-3')
a	RT-qPCR	Alpha-tubulin	TGAGGCCATTTACGACAT	AGATCACAGGAGCATAGGAG
		RrANT1	CTGGGGCTCTTCAACCCACA	CCACCTCCCATGCTGGTGAT
b	pBWA(V)HS-*ANT1*-GLosgfp expression vectors	*RrANT1*	cagt<u>CGTCTC</u>acaacatgaagcccatacatgatca	cagt<u>CGTCTC</u>atacaggtgtcattccaagcagcaa
c	overexpression vector construction	*RrANT*	GGACTCTTGA<u>CCATGG</u>TTATGAAGCCCATACATGATCACAGTC	GTCAGATCTA<u>CCATGG</u>GCCCTACTTTCCTCCTCCAT
d	DNA testing of transgenic plants	*RrANT*	AAGGAATGGTGCCGAACT	AGGGCTCATGGACAAAGTAA
		α-tubulin	TGGAGGATGGAAGGACTTTGG	CAGGACGACAACAAGCAACAG
e	gene fragment amplification	Hyg resistance gene	GTCCTGCGGGTAAATAGC	GGATCGGACGATTGCGT

Table S4 Genetic transformation medium for *Petunia hybrida*

Medium type	Formula
Infection solution	30g/L Sucrose + MS+0.5mg/L MES + 30μmol/L As
Pre culture medium	30g/L Sucrose + MS+7g/L Agar + 3.0mg/L 6-BA + 0.2mg/L IAA
Co-culture medium	30g/L Sucrose + MS+7g/L Agar + 6-BA 3.0mg/L + 0.2mg/L IAA + 30μmol/L As
Selection and differentiation medium	30g/L Sucrose + MS+7g/L Agar + 3.0mg/L 6-BA + 0.2mg/L IAA + 7mg/L Hyg + 500mg/L Cb
Screening rooting medium	30g/L Sucrose + 1/2MS + 7g/L Agar + 0.1 mg/L NAA + 6mg/LHyg + 500mg/L Cb

MS: Murashige & Skoog medium; MES: 2-Morpholinoethanesulfonic Acid; As: Acetosyringone; BA: 6-Biamido purine; IAA: Indole-3-acetic acid; Hyg: Hygromycin B; Cb: Carbenicillin sodium salt; NAA: Naphthaleneacetic acid

ATGAAGCCCATACATGATCACAGTCATAACAACAATGGAAGCCATATTAACAATAACAACAACTGGTTGGG
GTTTTCACTCTCAGCTCCCCACATGAAAATGGAGGTCACTTCTTCTTCTGACCCTCATCAACACTACAGTCA
TCAACAAGCTCAGGCCTCCTCTGCTTCAGCTCAGCTTCCAAGTAGCTTCTACAATCTGACCCCACTCTGCTA
TGAAAATGGTGGGTTTCACTCTCCCTTGACTGTAATGCCACTCAAGTCAGATGGGTCTCTTTGCATCATGGA
AGCTCTCACTCGCTCACAAGCTGAAGGAATGGTGCCGAACTCGTCCCCAAAACTTGAGGACTTTCTAGGA
GGAGCAAGCATGGGAGCTCATGACTATGGAACCCATCAAAGAGAGGTGGCAATGGCTCTCAGCTTAGACA
GCTTGTATTACAATGAAAATGCAGAGGCAGAGCAACAATACTACTCTGGAATCCCTTTCCCTGGAGCTTACC
AAACTCAAATGGAGGAAGAGTCCTCTAAGCAAGCCCACATTGTGGGCTGTGATAGTCACCAAATGACCCA
GAACTGGGTGACTAGGCAGTACTCTGCTGCTCATCATGCTTTCAATCAACAACACATGAGTAACAACAGCT
TGGACAATGGAGGAGTTTCTGGGTCTGTTAATGGTGGAATGCAGTGTGGGGATTTACAGTCTCTTACTTTGT
CCATGAGCCCTGGATCTCAGTCCAGCTGTGTCACAGCTCCAAGGCAGATCTCACCCACTGAAACAGAGAG
TCTAGCCATGGAAACCAAGAAGAGAGGTTCTGGTAAAATTAGTCAAACAAAGCAGCCAGTGCACAGAAA
GTCCATTGACACATTTGGGCAGAGAACTTCACAGTATAGAGGAGTCACAAGGCATAGATGGACCGGTAGAT
ATGAAGCTCATCTATGGGACAACAGTTGCAAGAAGGAGGGGCAAACCAGAAAAGGAAGGCAAGTTTATCT
TGGGGGATATGATATGGAAGAGAAAGCTGCAAGGGCATATGATCTTGCTGCTCTTAAGTACTGGGGCTCTTC
AACCCACATAAACTTCCCATTGGATGATTACACAACACAAATTGAAGAAATGAAGAATATGAACCGGCAAG
AGTATGTTGCGCATCTGAGGAGGAAAAGCAGTGGATTTTCAAGGGGAGCTTCAATTTACCGAGGAGTTACA
AGACATCACCAGCATGGGAGGTGGCAAGCTCGAATTGGCAGGGTTGCAGGAAACAAGGACCTTTATCTTG
GTACATTTGGCACCCAAGAGGAAGCAGCCGAAGCTTATGACATAGCTGCAATCAAGTTCCGTGGGGTAAAT
GCGGTCACCAACTTTGACATTACCAGATATGATGTTGAGAAAATCATGGCCAGCAATACTTTGCTTGCTGGA
GAATTCGCTAGGCGTAACAAGGAGGTTGAACCTAACAAACAAGTCATTGAGTACAACTCACCAGCACAGA
ACAATGTGGAAGCCAATACACCCGAAAATAGTCATGGGAATAGTTCAGATTGGAAAATGGCTTTGTACCAA
GCTGCACAGCAACAACAAGCAACTGCTGCTACTTGTGTTCAAACACTTGATCAAAAATCAATGTCTTCAGG
GAATTACAGAAGTCCTGCATCTTTCTCAATGGCATTGCAAGACCTCATTCGCGTTGAATCAGTGAACTCTAG
CCAGCAGTTGATGGATGAATCAGCTAAAGCCGGTGCTCATTTTTCAAATCCTTCGTCGCTGGTGACCAGTCT
GAGCAGCTCCAGAGAAGATAGCCCTGACAAATCAGGCCCCACAATGCTGTTTGCAAAACCTCCAATGGCA
TCAAAGTTCATAAGTCCAAGTACTGCTGCTGCTGTTAGCTCTTGGTTCCCATCAGCTCAGTTGAGGCCTGCA
GCTGCCATTTCCATGTCTCACTTGCCTCTATTTGCTGCTTGGAATGACACCTAG

Figure S1 The fragment of *RrANT1* amplified by PCR in the subcellular localization experiment

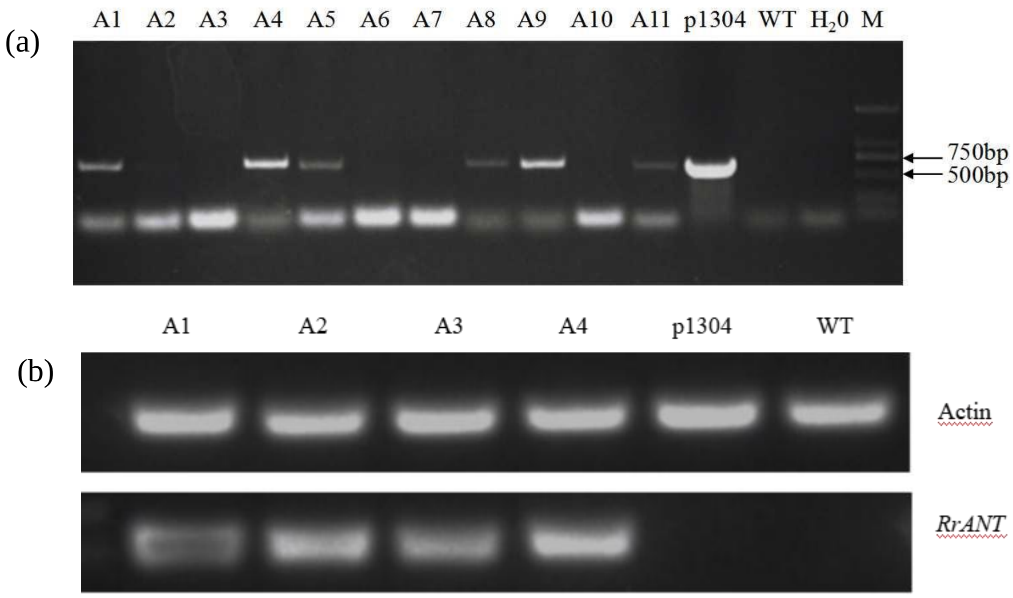

Figure S2 *RrANT1* verification and semi-quantitative analysis of transgenic petunias. (a) six of the eleven strains can amplify a single band with a length of ~750bp, while the wild type shows no band, which shows that the *RrANT1* gene has been integrated into the petunia genome. (b) The transgenic plants showed bands with brightness, indicating *RrANT1* had been successfully transferred into petunia and could be expressed,no bands were detected in the control groups.

A1-A11: overexpression petunias; p1304: petunias transformed into pCAMBIA1304; WT: wild Type; H2O: water; M: marker.
